# Because Doing “It” *Matters* to Patients: Development and Evaluation of a *What Matters to Me* Tool That Elicits Patients' Priorities to Support Cancer Treatment Shared Decision‐Making

**DOI:** 10.1002/cam4.71169

**Published:** 2025-09-11

**Authors:** Bruce D. Rapkin, Ariana E. Tao, Brieyona C. Reaves, Krystal A. Rivera, Lauren K. Jones, Rita R. Ravichandar, Dennis Yi‐Shin Kuo, Rafi Kabarriti, Alexander I. Sankin, Ahmed A. Aboumohamed, Kara L. Watts, Damara N. Gutnick, Ellen Miller‐Sonet

**Affiliations:** ^1^ Department of Epidemiology & Population Health Albert Einstein College of Medicine Bronx New York USA; ^2^ Cancer Clinical Trials Office Montefiore Medical Center Bronx New York USA; ^3^ Department of Obstetrics & Gynecology Division of Gynecologic Oncology Montefiore Medical Center Bronx New York USA; ^4^ Department of Radiation Oncology Montefiore Medical Center Bronx New York USA; ^5^ Department of Urology Montefiore Medical Center Bronx New York USA; ^6^ Department of Family and Social Medicine Albert Einstein College of Medicine Bronx New York USA; ^7^ Department of Psychiatry and Behavioral Sciences Albert Einstein College of Medicine Bronx New York USA; ^8^ CancerCare New York New York USA

**Keywords:** decision aid, patient clinician communication, patient values, patient‐centered care, shared decision‐making, what matters to patients

## Abstract

**Introduction:**

Cancer impinges on nearly every aspect of the lives of patients, survivors, and loved ones. This study presents progress in developing the “What Matters to Me” Worksheet (WMTM‐Worksheet), designed to elicit personal priorities across multiple life domains. WMTM‐Worksheet items were finalized based on clinician recommendations and patient feedback. Individuals at any point in cancer treatment were interviewed post‐appointment about using the WMTM‐Worksheet prior to their appointment.

**Methods:**

To finalize the WMTM Worksheet, initial samples of clinicians and patients were interviewed on its content and usability. Oncology clinicians were recruited by email; 25 accepted and were surveyed about current practices of incorporating patient priorities and preferences into treatment planning, and the usability, practicality, and feasibility of the 17‐item WMTM Worksheet. Patients were English‐ or Spanish‐speaking adults diagnosed with gynecological, head and neck, or urological cancers. Patients at any point in active treatment or follow‐up were eligible. An initial sample of 15 patients was administered a cognitive interview about the WMTM Worksheet by telephone to assess its clarity, relevance, and feasibility. Next, 61 patients taking part in the user experience portion of this study were identified through the electronic medical record (EMR), contacted by telephone, and offered participation in a onetime interview regarding the WMTM Worksheet. They received the WMTM Worksheet prior to a clinic appointment. Questions regarding user experience were administered by telephone 1–3 days later.

**Results:**

Of the 61 patient respondents, 57% were over age 65, 59% female, 41% Hispanic, 45% Black; 49% had an annual income below $35,000. Patient responses yielded seven principal components, reflecting domains such as symptoms, family caregiving, work, and hobbies. Most patients (62%) said the WMTM‐Worksheet helped them think about disease and treatment; 30% said it helped communication with clinicians. Eighty‐five percent were glad to share their concerns, and only 10% found it difficult to complete.

**Conclusion:**

The WMTM‐Worksheet can bring patients' broader priorities into care planning. Patients may be better able to anticipate and avoid problems. Discussion of priorities validates patients' concerns and promotes trust. Implementation will require the clinical infrastructure to support shared decision‐making and incorporate the WMTM‐Worksheet into workflow. Oncologists may benefit from communications training to determine patients' concerns and present options that best address their priorities.

## Graphical abstract

This study evaluated the “What Matters to Me” Worksheet, a tool designed to elicit personal priorities across multiple life domains. Patients undergoing cancer treatment reported the WMTM Worksheet helped them communicate with their clinician and think about their disease and treatment.

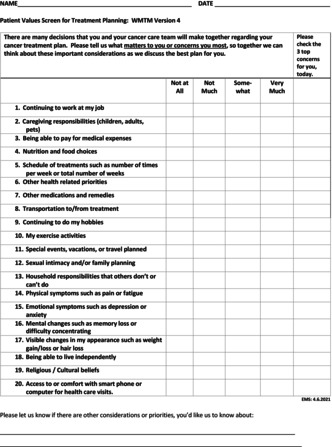

## 1 Introduction

Patient: *“Doctor, it hurts when I do this.”*

Doctor: *“If it hurts, then stop doing it.”*

Patient: *“But doing ‘it’ is so important to me.”*

What if doing “it,” be it lifting a grandchild, working, dancing, playing piano, having sex, taking a family vacation, or cooking holiday dinner, really matters to the patient? The intensity, timing, and side effects of cancer and its treatment can have implications for patients' daily activities and their longer term personal goals and quality of life. Incorporating an understanding of what matters to patients into treatment planning can enable effective person‐centered treatment planning and shared decision‐making (SDM) [1]. With information about individuals' life circumstances and personal concerns, oncology clinicians may be better equipped to help patients anticipate, manage, and at times avoid undesirable consequences. Yet when decision‐making criteria primarily center on treatment efficacy and side effects, implications for roles and activities most important to a given individual can get lost.

This study presents progress in the development of the “What Matters to Me” Worksheet (WMTM‐Worksheet), a one‐page form designed to elicit patients' priorities across a broad range of life domains. It is intended for use in clinical practice to prompt conversation about patients' broader circumstances and concerns. By understanding *what matters* to individual patients, we hypothesize that oncologists will be better able to recommend treatments that minimize burden, avoid unintended consequences and support quality of life. By framing discussion in terms of impact on patients' priority activities and goals, potential barriers to self‐care or treatment adherence may be easier to anticipate, recognize and address [2]. Acknowledging patients' personal concerns is also likely to promote greater patient satisfaction with care and trust of providers [3].

The Institute of Medicine, an American nonprofit nongovernmental organization, published their report, *Crossing the Quality Chasm*, in 2001 that established “patient‐centeredness” as intrinsic to high‐quality care [4, 5]. Patient‐centeredness includes respect for patient values, preferences, and expressed needs, along with patient education and materials that are easily understood [6, 7]. Such patient‐centered practices improve patient satisfaction and safety [8], and correlate with better health outcomes [9] often with lower costs [10].

In cancer, research on SDM using decision aids shows it has the potential to improve patient understanding and information retention, patient and family engagement, and promote decisional satisfaction and adherence [11, 12, 13, 14, 15, 16, 17]. Decision aids have also highlighted structural barriers [18], contributing to reduced conflicts with patients and greater clinician satisfaction [19]. However, cancer decision aids have generally focused on the clinical aspects of illness and treatment, such as self‐reported symptoms, treatment risks and benefits, preferences for SDM, and decisional conflict. Patients' broader priorities and values are generally not addressed [20]. In a scoping review of 11 high‐quality SDM studies in the United States [21], only one study included assessment of patient values [22]. Despite this lack of attention, evidence suggests that cancer patients want to have conversations about personal concerns with clinicians [23]. In a 2018 survey of 320 patients seeking services from Cancer*Care* (a US‐based national support and advocacy organization), 62% said it was important that clinicians understand their priorities. Only 37% reported having such discussions prior to treatment [24, 25].

Over the past decade, emphasis on asking *what matters* to individuals has emerged as foundational to high‐quality patient‐centered care [7, 26]. A 2012 NEJM article [7] inspired the birth of the What Matters to You (WMTY) movement. The Institute for Healthcare Improvement (IHI) challenged healthcare providers to shift their focus from “What's the matter?” to “What matters to you?” This movement quickly gained traction, spreading to over 50 countries and five continents [27]. Growing evidence supports its impact on care outcomes, utilization, staff joy in work and patient experience [7, 28, 29, 30, 31, 32, 33, 34, 35, 36].

It is instructive to distinguish how assessment of what matters to patients as envisioned by the IHI differs from psychosocial distress screening in oncology practice. Distress screening is also a key component of person‐centered cancer care. Since 2015, the Commission on Cancer, a consortium of professional organizations established by the American College of Surgeons, has required institutions to routinize screening for distress and unmet needs as part of its accreditation process [37, 38]. The widely adopted NCCN distress thermometer and problem list, as well as social determinants screening [39] are designed to identify problems that patients are currently experiencing, prompting referral to services. Screening and referrals are intended to identify and address emotional issues and barriers to care, but they are not intrinsically part of treatment planning.

In contrast, the proactive assessment of *what matters* is intended to identify patient priorities and circumstances that may subsequently be affected by treatment choices. Rather than an exclusive focus on problems, the goal is to identify the importance of different aspects of life. With this information, clinicians and patients can weigh the implications of treatment options considering patients' unique priorities. Treatment plans can then consider and reflect individual circumstances, perhaps even preventing distress. At present, oncology clinicians do not routinely elicit such information due to concerns that include the time and resources required for comprehensive decision support [24, 40]. The challenge is to introduce an efficient and effective way to collect and introduce this information into the processes of treatment planning and SDM.

## 2 Materials and Methods

### 2.1 Development of the Initial WMTM‐Worksheet

The WMTM‐Worksheet was developed by Cancer*Care*'s Patient Values Initiative Advisory Board. Composed of patient advocates and oncology experts (Hector Nunez, BS, Patient Advocate and Survivor; Amy Berman, BSN, LHD, Patient Advocate and Survivor; Patricia Goldsmith, CEO, Cancer*Care* and Survivor; Patti Jewell, BA, MPA, Cancer*Care* Board of Directors; Edith P. Mitchell, MD, FACP, Sidney Kimmel Cancer Center, Thomas Jefferson University; Lee Schwartzberg, MD, FACP, The West Clinic; Mike Zincone, BS, Pfizer Oncology; Randy Burkholder, MBA, Pharmaceutical Research & Manufacturers of America; Mike Wong, MD, PhD, FRCPC, University of Texas, MD Anderson Cancer Center; Susan Love, MD, MBA, Dr. Susan Love Research Foundation; Ellen Miller‐Sonet, MBA, JD, Cancer*Care*; Sandy Kurtin, RN, MS, AOCN, ANP‐C, University of Arizona Cancer Center; Cynthia Manley, Digital Strategist, Vanderbilt Medical Center), the group was convened as part of a new initiative seeking to reframe the national healthcare policy dialog to include what is important to patients and their families, and to incorporate patients' values and priorities into treatment decision‐making models [41]. The WMTM‐Worksheet was designed to facilitate conversation between patients and clinicians regarding patients' quality of life priorities for use during treatment planning and at points of transition in care. Through much discussion and iteration, the Board agreed to include 17 life priorities often cited by patients with cancer. The choice of these factors was based on their personal experiences as patients and clinicians. The list was then exposed to Cancer*Care* clients via an online survey to confirm their importance. In this unpublished survey, the responses of 289 confirmed cancer patients supported the relevance of these priorities on a five‐point scale of “important or unimportant to you as a person living with cancer”. The survey probed issues that may not have “made the list”, and none were identified.

On the WMTM‐Worksheet, priorities rated on a four‐point importance scale from ‘Not at All’ to ‘Very Much’ included work, caregiving responsibilities, transportation, managing comorbidities and relationships that contribute to individuals' quality of life. After rating the importance of each area, the WMTM‐Worksheet also asked respondents to identify their three most important concerns.

### 2.2 Study Setting

Cancer*Care* and the Montefiore Einstein Comprehensive Cancer Center (MECCC) collaborated on this study. MECCC serves the Bronx, NY, with a population of nearly 1.4 million that is 55% Hispanic/Latinx ethnicity, 45% Black, 9% white non‐Hispanic, and 4% two or more races [42]. Bronx residents are 34% foreign‐born, and 58% speak languages other than English, predominantly Spanish [42]. More than 32% of residents report annual household income below $25,000 [43]. Although overall cancer incidence is 9% lower than New York State, mortality is 10% higher [44].

The aims of this study were (1) to elicit recommendations from clinicians concerning the use of the WMTM‐Worksheet; (2) to obtain feedback from patients regarding the clarity, usability, and relevance of the WMTM‐Worksheet; and (3) to examine cancer patients' experience using the WMTM‐Worksheet. The study was reviewed and approved by the Einstein Institutional Review Board (#2020–12074) prior to all study activities.

### 2.3 Aim 1: Clinician Review and Recommendations

Eligible clinicians were those who treat adult cancer patients at MECCC. 111 eligible clinicians were asked to participate in this research via email outreach. Twenty‐five (23%) provided consent and completed an online survey about current practices of incorporating patient priorities and preferences into treatment planning and the usability, practicality, and feasibility of the 17‐item WMTM‐Worksheet.

### 2.4 Patient Eligibility

For Aims 2 and 3, eligible patients were English‐ or Spanish‐speaking adults diagnosed with gynecological, head and neck, or urological cancers. Patients at any point in active treatment or follow‐up were eligible. We chose to focus on these three diseases to reach patients who varied by cancer burden, as well as by sex and age.

### 2.5 Aim 2: Cognitive Interviews

To ensure clarity of the WMTM‐Worksheet, 15 patient interviews were conducted, 10 in English and five in Spanish. Eligible patients of participating clinicians were identified through the electronic medical record (EMR), contacted by telephone, and offered participation in a onetime interview regarding the WMTM‐Worksheet. Patients were asked open‐ended questions regarding the content and usability of the WMTM‐Worksheet. Verbal informed consent was obtained. A $25 gift card was provided at interview completion.

### 2.6 Finalization of the WMTM‐Worksheet

**FIGURE 1:**
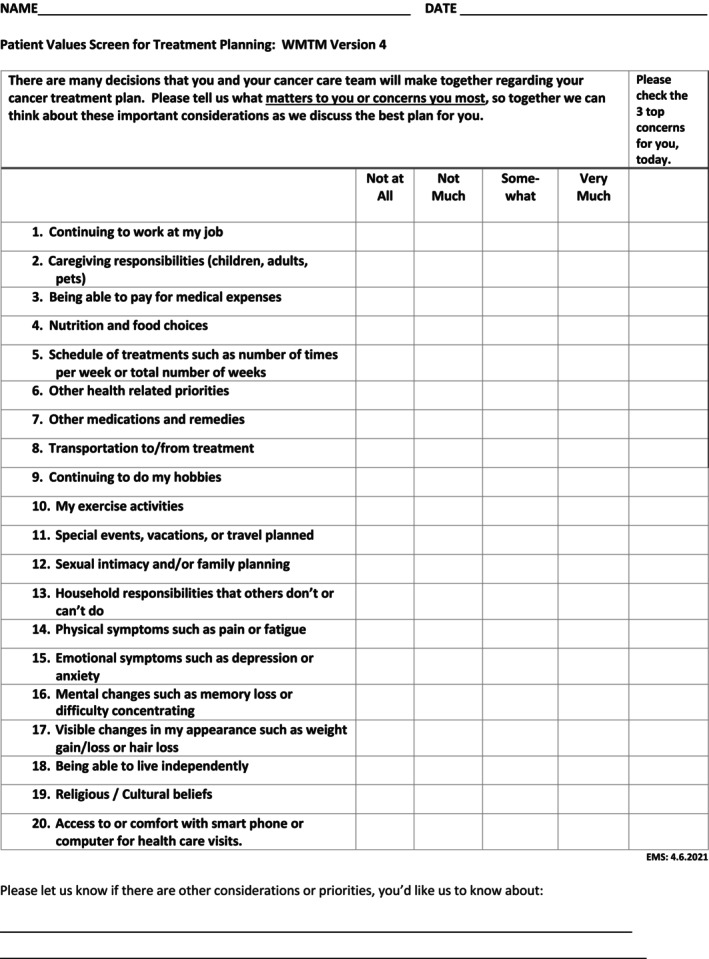
Finalized 20‐item What Matters To Me (WMTM) Worksheet.

After formative work in Aims 1 and 2, three items were added based on clinicians' and patients' suggestions: Nutrition and food choices, Other medications and remedies, and Other (non‐cancer) health priorities (Figure 1).

### 2.7 Aim 3: Patient Recruitment Procedures

Referencing the EMR between August and November 2022, 433 eligible patients were identified. The WMTM‐Worksheet was offered to 159 (37%) patients in clinic prior to their oncology appointment. Of these, 127 (80%) accepted and completed the WMTM‐Worksheet. We did not routinely share completed WMTM‐Worksheets with clinicians for this study; but patients could choose to do so.

Sixty‐one patients (20 patients had gynecological, 21 head and neck, 20 urological cancer) who completed the WMTM‐Worksheet in clinic participated in the survey. To achieve this sample, 111 of 127 (87%) patients who completed the WMTM‐Worksheet were called and offered participation in the survey. We were unable to reach 40 of the 111 (36%) patients after three attempts. Of the 71 patients (64%) we reached, 10 (14%) declined participation, and 61 (86%) provided informed consent to participate in the follow‐up survey by telephone. Eleven of 61 interviews (18%) were conducted in Spanish. All survey participants received a $25 gift card.

### 2.8 Aim 3: Survey Instrument Description and Procedures

A survey of patients' experiences using the WMTM‐Worksheet addressed recollection and ease of use (5 items) and discussion with clinicians (3 items). Ten additional items assessed patients' feelings about completing the WMTM‐Worksheet and their clinician's consideration of their personal priorities. Demographic information collected included sex, race, ethnicity, years in the US, primary language, living situation, household income, and health insurance coverage. Date of diagnosis obtained from the EMR was also considered.

### 2.9 Statistical Analysis

Analysis included descriptive statistics, principal component analysis to summarize sets of items, and correlations to examine relationships among participant characteristics, personal concerns identified, and experience using the WMTM‐Worksheet. Analyses used pairwise deletion for sporadic missing data. Significant bivariate results (*p* < 0.05, two‐tailed) are reported in text. Tables also indicate trends (*p* < 0.10, two‐tailed). Analyses were conducted using IBM SPSS Statistics (version 29).

## 3 Results

### 3.1 Preliminary Evaluation of WMTM‐Worksheet

#### 3.1.1 Aim 1: Clinician Review and Recommendations

Regarding current practice, 16 of 25 clinicians indicated that they discuss patients' personal priorities most or all of the time, with 15 stating that they did so before initiating treatment. The remaining nine said that they discussed patient priorities prior to treatment at least occasionally. Among all participating clinicians, 19 of 25 stated that they documented discussion of patients' priorities most or all of the time, with information routinely shared with the clinical team through the EMR (11 of 25), at team meetings (10 of 25), by emails to the team (7 of 25), and through informal conversation (13 of 25). Sixteen of 25 clinicians reported that patient priorities affect their treatment recommendations a lot or always.

As Table 1 shows, 12 of the 17 initial WMTM‐Worksheet priority areas were rated as important to patients by at least 22 of the 25 clinicians. Only two areas received lower clinician priority ratings: Exercise (13 of 25) and Access to or Comfort with Smartphone or Computer for Healthcare Visits (13 of 25). Clinicians also proposed areas to add to the WMTM‐Worksheet, including body image, financial challenges, change in prognosis, end‐of‐life planning and healthcare proxies, short‐ and long‐term treatment side effects, alternative medicine options, engaging family members who live outside of the region, and tradeoffs between treatment efficacy versus quality of life.

**TABLE 1:** Areas of patient concern deemed important by clinicians (*N* = 25).

	*N*
Continuing to work at my job	24
Transportation to/from treatment	24
Special events or vacations planned	24
Physical symptoms such as pain or fatigue	24
Visible changes in my appearance such as weight gain/loss or hair loss	24
Caregiving responsibilities	23
Schedule of treatments	23
Household responsibilities that others don't or can't do	23
Being able to live independently	23
Being able to pay for medical expenses	22
Emotional symptoms such as depression or anxiety	22
Mental changes such as memory loss or difficulty concentrating	22
Sexual intimacy and/or family planning	21
Continuing to do my hobbies	20
Religious/cultural beliefs	19
My exercise activities	13
Access to or comfort with smart phone or computer for healthcare visits	13

Most clinicians found the WMTM‐Worksheet to be useful (23 of 25), beneficial to communication (23 of 25), and patient‐friendly (20 of 25). Only 3 of 25 believed the form was redundant or unnecessary; however, 6 of 25 were concerned that the form could be confusing. Clinicians believed that the Worksheet could be useful at new patient visits (16 of 25), prior to such visits (6 of 25), and during treatment transitions (10 of 25). Despite this positive reception, 13 of 25 clinicians remained unsure whether they would use the WMTM‐Worksheet in their practice; 8 of 25 clinicians anticipated that it would be somewhat or very difficult to implement into clinic workflow.

#### 3.1.2 Aim 2: Cognitive Interviews With Patients

We contacted 23 patients by phone between January and July 2021; 15 agreed and eight declined to participate in a onetime interview. Table 2 shows the characteristics of patients who were offered participation. We desired a representative group of participants for the interview. Nearly all participants were either Black (7 of 15) or Hispanic/Latino (7 of 15). Patients who declined were mostly Hispanic/Latino (6 of 8). Participants were equally disturbed in terms of cancer type (five of each head and neck, gynecological, or urological) and cancer stage, with 7 of 15 having early stage disease and 7 of 15 later stage. As is common practice in qualitative research, cognitive interview questions were modified progressively to consider information gained. The first two patients were interviewed about phrasing and readability of the WMTM‐Worksheet. Since both displayed adequate understanding, the subsequent 13 patients were interviewed about usability, content, and purpose. Most respondents believed the WMTM‐Worksheet would be a useful way for patients to communicate with doctors about personal concerns and suggested that it be implemented when discussing treatment. Most respondents had positive feedback, and only one patient expressed pessimism regarding the value of sharing this information.

**TABLE 2:** Characteristics of patients offered participation in Aim 2 cognitive interview.

	Participated	Declined	Total
**Cancer type**			
Head and Neck	5	4	9
Urological	5	1	6
Gynecological	5	3	8
**Cancer stage**			
I	5	3	8
II	2	0	2
III	5	1	6
IV	2	3	5
Unknown	1	1	2
**Race**			
African American/Black	7	1	8
White	2	1	3
Other	6	5	11
Unknown	0	1	1
**Ethnicity**			
Hispanic/Latino	7	6	13
Not hispanic/Latino	8	2	10

### 3.2 Aim 3: Patient Evaluation of the WMTM‐Worksheet

#### 3.2.1 Participant Characteristics

The sixty‐one participants are described in Table 3. The sample is predominantly low‐income, African American/Black, and Hispanic/Latinx, with 50% non‐US born, representative of MECCC's patient population. Sample variation in US nativity, primary language, living situation, and socioeconomic measures made it possible to examine their potential associations with personal concerns and WMTM‐Worksheet experiences.

**TABLE 3:** Aim 3 participant characteristics.

		*N*	Total *N*	%
Cancer diagnosis	Gynecological	20	61	33%
	Head and Neck	21	61	34%
	Urological	20	61	33%
	Diagnosed < 1 year ago	33	61	54%
Demographics	Age ≥ 65	35	61	57%
	Female	36	61	59%
	Hispanic/Latinx	25	61	41%
	African Descent/Black	26	58	45%
	White	13	58	22%
	Multiracial	12	58	21%
Immigration history	Born in the United States	30	60	50%
	Spanish is first language	19	61	31%
	English with provider—comfortable	15	20	75%
	English with provider—very comfortable	9	20	45%
	Interviewed in Spanish	10	61	16%
Living situation	Do not rent or own	19	61	31%
	Rent where I live	19	61	31%
	Own where I live	22	59	37%
	Live with spouse/partner	28	60	47%
	Live with other adult	36	61	59%
	Live with minors	13	61	21%
Socioeconomic status	High school education or less	28	59	47%
	Vocational/technical training	29	59	49%
	Employed full‐ or part‐time	18	59	31%
	Unemployed	10	59	17%
	Retired	22	59	37%
	Disabled	13	59	22%
Household income	More than one contributor to income	25	61	41%
	Annual income > $35,000	23	47	49%
	Getting by or comfortable with income	39	61	64%
Insurance	Medicare	23	60	38%
	Medicaid or dual	24	60	40%
	Other insurance	13	60	22%

#### 3.2.2 Ratings of What Matters to Patients

**FIGURE 2:**
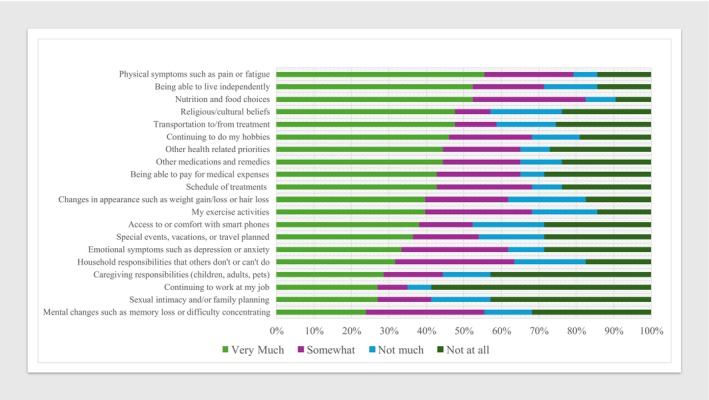
Patient ratings of importance of WMTM‐Worksheet items (*N* = 63).

Figure 2 summarizes patients' ratings of *what matters* to them, from 1 (Not at all) to 4 (Very Much). Each WMTM‐Worksheet domain was important to a sizable proportion of respondents. Areas most frequently rated as mattering “Very Much” included Nutrition, Physical Symptoms, and Living Independently. All items except Intimacy, Work, and Caregiving were rated as mattering “Very Much” or “Somewhat” by over 50% of respondents.

#### 3.2.3 Patterns of Association Among WMTM‐Worksheet Priority Ratings

Principal components analysis (PCA) summarized patterns of concerns (Table 4). Seven orthogonal components accounted for 71% of total variance. Patterns of concerns included: physical and emotional symptoms; management of non‐cancer health conditions; maintaining activities and keeping up with responsibilities; concerns related to spirituality and staying independent versus work; caregiving and special events; nutrition and treatment costs; and intimacy.

**TABLE 4:** Principal components of patient concerns on WMTM‐Worksheet items.

	Principal components							Item communalities
	1	2	3	4	5	6	7	
	Physical and emotional symptoms	Managing health conditions	Activities and responsibilities	Cultural and connected	Caregiving and special events	Nutrition and treatment costs	Intimacy	
Mental changes such as memory loss or difficulty concentrating	**0.738**	0.210	−0.020	0.246	0.125	−0.147	−0.075	0.693
Physical symptoms such as pain or fatigue	**0.722**	0.139	0.193	−0.176	0.060	0.113	0.300	0.716
Visible changes in my appearance such as weight gain/loss or hair loss	**0.710**	0.078	0.119	0.222	−0.096	0.187	0.026	0.619
Emotional symptoms such as depression or anxiety	**0.567**	0.382	0.096	−0.054	0.300	0.032	−0.289	0.653
Other health related priorities	0.152	**0.896**	0.049	0.099	0.071	0.061	0.035	0.848
Other medications and remedies	0.121	**0.873**	−0.019	0.024	0.137	0.087	0.174	0.834
Transportation to/from treatment	0.287	**0.595**	0.430	0.060	−0.222	0.287	−0.040	0.757
Schedule of treatments such as number of times per week or total number of weeks	0.147	**0.549**	0.159	−0.006	0.182	**0.500**	−0.131	0.648
Continuing to do my hobbies	0.009	0.099	**0.834**	0.109	0.136	0.079	−0.012	0.743
My exercise activities	0.196	0.018	**0.758**	−0.006	0.048	−0.032	0.301	0.707
Household responsibilities that others don't or can't do	0.466	0.073	**0.575**	−0.036	0.421	0.020	−0.070	0.736
Religious/cultural beliefs	0.168	−0.063	0.038	**0.832**	−0.040	0.106	−0.180	0.772
Access to or comfort with smart phones	−0.130	0.221	0.342	**0.616**	−0.125	0.076	0.199	0.623
Being able to live independently	0.364	−0.058	0.261	0.443	−0.171	0.377	0.333	0.682
Caregiving responsibilities (children, adults, pets)	0.042	0.133	0.119	−0.081	**0.816**	0.252	0.068	0.774
Special events, vacations, or travel planned	0.294	0.111	0.388	−0.078	**0.520**	0.052	0.430	0.714
Nutrition and food choices	0.027	0.288	−0.067	0.084	0.206	**0.704**	0.059	0.637
Being able to pay for medical expenses	0.436	0.038	0.120	−0.164	0.276	0.424	0.139	0.508
Continuing to work at my job	−0.068	−0.147	0.294	−0.669	−0.025	0.419	0.118	0.750
Sexual intimacy and/or family planning	0.010	0.084	0.116	−0.073	0.098	0.054	**0.875**	0.804
Eigenvalues of rotated components	2.749	2.650	2.344	1.940	1.534	1.501	1.500	
Variance explained by rotated components	0.137	0.133	0.117	0.097	0.077	0.075	0.075	

*Note:* Coefficients greater than 0.50 were bolded in the original Table 4. Green highlighted text indicates primary coefficients that define each principal component. Yellow highlighted text indicates secondary coefficients that load on more than one principal component. Summary statistics are presented in gray rows and columns.

#### 3.2.4 Patients' Evaluation of the WMTM‐Worksheet

Table 5 summarizes participants' experiences completing the WMTM‐Worksheet.

- *Ease of Completion*: Most (82%) immediately recalled completing the WMTM‐Worksheet; after being prompted to recall the WMTM‐Worksheet if necessary, 90% of all respondents indicated it was not difficult to complete.
- *Facilitation of Communication*: Thirty percent of participants indicated the WMTM‐Worksheet helped them communicate with clinicians. This was about twice as likely among people with high school education or less (*χ* ^2^ = 2.73, 1 df, *p* < 0.10).
- *WMTM‐Worksheet Helps Patients to Consider Priorities*: About 85% were glad to share personal priorities with clinicians, and over half (58%) felt relieved to disclose. Most (62%) said the WMTM‐Worksheet helped them think about how treatment could affect their lives, especially patients diagnosed more recently (*r* = −0.36, *p* < 0.01), female patients (72% vs. 48%, *χ* ^2^ = 3.69, 1 df, *p* < 0.06) and Black and Latinx patients (71% vs. 45%, *χ* ^2^ = 3.79, 1 df, *p* < 0.052).
- *Distressed Patients' Experience of Using the WMTM‐Worksheet*: Small proportions of patients said they felt stressed (29%) or scared (19%) when completing the WMTM‐Worksheet. Feeling stressed was associated with concerns about upcoming travel and events (*r* = 0.36), caregiving (*r* = 0.32), household responsibilities (*r* = 0.30), and symptoms (*r* = 0.32). Feeling scared was correlated with cognitive concerns (*r* = 0.39). Notably, patients reporting stress or anxiety (34%) were significantly more likely to indicate that the WMTM‐Worksheet helped them think about personal priorities (*χ* ^2^ = 5.60, 1 df, *p* < 0.02).

**TABLE 5:** Patient evaluation of the WMTM‐Worksheet.

		*N* agreed	*N* total	% agreed
Discussion of WMTM‐W	Remember the WMTM‐W?	50	61	82%
	Doctor explained WMTM‐W	8	61	13%
	Doctor discussed WMTM‐W when reviewing options	13	61	21%
Relationship with Doctor	Doctor considers what is important to me	43	60	72%
	Doctor cares about me	44	59	75%
	Doctor is invading my privacy	1	59	2%
Feelings completing WMTM‐W	Stressed	17	59	29%
	Scared	11	59	19%
	Confused	13	59	22%
	Overwhelmed	15	58	26%
	Tired	17	58	29%
	Relieved	30	59	51%
	Glad to share	49	58	85%
Evaluation of WMTM‐W	Difficult to fill out	6	61	10%
	Helped about how treatment could affect my life	38	61	62%
	Helped communicate concerns about cancer treatment	18	61	30%

## 4 Discussion

### 4.1 Summary of Findings

Both patients and clinicians found the WMTM‐Worksheet to be valuable. Patients readily understood and appreciated its purpose. Clinicians agreed that knowing patients' concerns was important to treatment planning and care management.

Patients were positive about completing the WMTM‐Worksheet and sharing information with clinicians. Only 10% found it difficult to complete. Nearly two‐thirds said it helped them think about how treatment could affect their lives. This was especially the case among those feeling stressed or anxious, those closer in time to their diagnosis, and those concerned about physical and emotional symptoms. Nearly one‐third reported that the WMTM‐Worksheet helped them communicate with clinicians. Those not comfortable speaking English were more willing to use the WMTM‐Worksheet to share concerns.

Most clinicians found the WMTM‐Worksheet to be patient‐friendly, non‐redundant, and understandable. All but one believed it would enhance communication. Most said a tool such as this was needed to better understand patients' priorities. However, about half were unsure whether they would use the WMTM‐Worksheet in practice. Nearly one‐third anticipated difficulty in implementation due to workflow.

### 4.2 Preliminary Evidence for the Validity of the WMTM‐Worksheet

From a psychometric perspective, principal components and correlational analyses suggest that the WMTM‐Worksheet is capturing meaningful information. The full range of scale response options was used and missing data was minimal. Distributions differed across items, indicating that some concerns were more widely shared than others. Worksheet items were answered in an internally consistent manner, with people responding in similar ways to related concerns such as symptoms or levels of activity. Although replication in a larger sample is needed, principal component analysis findings make sense clinically and psychologically.

Association of concerns with demographic variables was readily interpretable. As is often the case with self‐reported measures, females reported higher levels of distress than males [45]. Individuals concerned about work were relatively younger, employed, and lived with minor children. These findings support our contention that individuals come to cancer treatment with priorities and concerns reflecting different life circumstances that can be readily assessed and considered in treatment planning.

### 4.3 The Role of the WMTM‐Worksheet in Shared Decision‐Making

With SDM as the gold standard of care, patient decision aids (PDAs) are promising tools. As an adjunct to disease‐ and treatment‐specific PDAs, we expect that the WMTM‐Worksheet would make the process of shared decision‐making even more useful and meaningful to patients. Deeper discussion of options in the context of patients' quality of life concerns should enhance patients' satisfaction with the relevance of health information [46]. Further, taking time to elicit and discuss patients' concerns signals that the care team values patient input.

### 4.4 “What Matters to Me” in Health Care for the Medically Underserved

The WMTM approach has particular relevance to cancer care in medically underserved communities. As noted, patients not comfortable speaking English were more willing to share concerns through the WMTM‐Worksheet. The WMTM‐Worksheet may interrupt implicit biases in clinical encounters by challenging clinicians' assumptions and stereotypes about individuals' life circumstances and personal goals. Conversations prompted by the Worksheet can help patients feel heard as individuals, building trust and promoting satisfaction with care.

### 4.5 Limitations

At this stage, we were unable to incorporate the WMTM‐Worksheet into the EMR, so any discussion of the tool had to be initiated by patients. As such, only one in five patients discussed the WMTM‐Worksheet with their doctor. We did not observe patient‐clinician interactions using the WMTM‐Worksheet. This study was also limited in terms of our focus on three different types of cancer. Our decision to sample three distinct patient groups was not predicated on the need to make comparisons by disease. Rather, we wanted a sample of patients with a mix of different concerns. Although research on the concerns of a much broader range of cancer patients is warranted, we expect that there will be greater similarities among patients in like situations or facing common challenges. Concerns may differ among patients based on disease and treatment characteristic; for example, further research is needed into patients whose diseases are harder to treat or who have greatly reduced life expectancy. Concerns may also depend upon disease‐specific functional deficits not considered in this study, such as mobility limitations or compromised immunity. Concerns may differ among people with rare cancers, early onset disease, or diseases with high familial risk.

The original impetus for developing the WMTM‐Worksheet was to prompt both patients and providers to consider patients' non‐disease‐related concerns in treatment planning and decision making. Although our initial plan was to provide patients with the WMTM‐Worksheet immediately prior to a conversation involving a treatment decision, this proved impractical. It was difficult for us to anticipate when such discussions would occur in the course of ongoing care. Even with newly diagnosed patients, it was difficult to fit administration of the WMTM‐Worksheet into the sequence of team‐based care. It will be more feasible to examine this when assessment of concerns can be incorporated as a scheduled component of initial treatment planning. Even so, there were also advantages in offering the WMTM‐Worksheet to patients at any point in their course of treatment. Patients' circumstances and concerns can and do change unpredictably, warranting reconsideration of earlier plans and introduction of new resources.

Although patients who were not at a decision point were less likely to discuss the WMTM‐Worksheet with providers on the day they received it, they did indicate its potential for helping them to think through concerns and for facilitating communication. Ideally, the issues raised by the WMTM‐Worksheet could and should be revisited by patients and physicians any time they become relevant throughout the course of care. Future longitudinal research will help to determine how this can best be achieved through timing and repetition of administration, communication prompts, and communication skills training.

Had a patient been at a point when a treatment decision was pending, we expect the WMTM‐Worksheet would have prompted discussion regarding how treatment options might impact the patient's quality of life priorities. This would allow the patient to participate more fully in shared decision‐making through an understanding of how treatment could affect their life.

### 4.6 Implications

Including patients in decisions about their health care is an ethical imperative [47]. In 1982, the President's Commission for the Study of Ethical Problems in Medicine publicly recognized the need to ensure meaningful *informed consent* for routine treatment through SDM. Some progress has been made toward adoption of this philosophy. Despite widespread consensus of its importance, cancer patients' personal preferences and values are not routinely considered in discussions of treatment [48]. Although the use of disease or treatment‐specific PDAs may only be warranted when patients face a complicated or difficult choice, attention to what matters to individuals is relevant to virtually all discussions of cancer treatment. Indeed, with limited time, physicians may be challenged to ensure that patients partake in the decision‐making process, especially when the treatment choice is a forgone conclusion [46]. The WMTM‐Worksheet provides a tool that is relatively easy to administer and interpret to ensure that discussions are person‐centered. We expect that this approach will yield benefits similar to other patient engagement strategies: greater adherence to treatments and appointments; more timely and accurate reporting of emergent symptoms; fewer emergency visits and subsequent hospitalizations; greater retention in follow‐up care posttreatment; higher satisfaction with care and greater quality of life [49, 50, 51, 52].

### 4.7 Next Steps

To realize its benefits in the broader oncology patient population, implementation of the WMTM‐Worksheet will require changes in clinical workflow [53]. Ideally, the WMTM‐Worksheet would be completed by patients in advance of their first oncology visit and updated at treatment transition points. The completed WMTM‐Worksheet could be considered by the care team before the visit to better integrate patient concerns into treatment planning. Patients' introduction to their clinical team should also clarify which team members can help with the specific concerns that they mentioned, reinforcing the idea that patient‐centered care is based on relationships. An app or a patient portal survey that feeds into the EMR would facilitate sharing across the team.

In situations requiring shared decision‐making regarding specific treatment choices, it might be plausible to incorporate the WMTM‐Worksheet into a preliminary PDA information packet. Subsequent in‐depth discussions of treatment decisions would be guided by this initial introduction. It would be important to provide clinicians with communication training and resources to help them respond to patients' concerns. Lay navigators could also play an integral role by helping patients formulate questions for WMTM conversations with clinicians.

Tinetti and colleagues describe the implementation of a Patient Priorities Care Initiative in US clinics to better meet the needs of patients living with multiple chronic conditions [54]. Their iterative, three‐step process involves patient values clarification and goal setting, development of treatment options, and SDM to establish a treatment plan. Clinicians were trained to employ this model by a Patient Priorities implementation team of experienced clinicians and stakeholders. This work could provide a helpful prototype for oncology.

Future research is needed to examine the effectiveness of the WMTM‐Worksheet in clinical practice. Examining the use of the Worksheet in real‐world practice would provide important evidence to support wider use. Research is also needed to understand how to implement the tool with different patients in varied clinical oncology settings. We expect that cluster‐randomized trials would be the best way to obtain such evidence; because full implementation would require training of oncology teams, modification of workflows, and integration with information systems at a clinic‐wide level. Strategies to align use of the tool with the needs of particular patients and clinicians may support and encourage greater use. If warranted by future research, practice guidelines should incorporate the use of the WMTM‐Worksheet to emphasize systematic consideration of individual patients' priorities to inform shared treatment decisions and to optimize patients' quality of life.

## 5 Conclusion

The WMTM‐Worksheet can bring patients' broader priorities into care planning. Patients may be better able to anticipate and avoid problems. Discussion of priorities validates patients' concerns and promotes trust. Implementation will require the clinical infrastructure to support shared decision‐making and incorporate the Worksheet into workflow. Oncologists may benefit from communications training to determine patients' concerns and present options that best address their priorities.

## Author Contributions

**Bruce D. Rapkin:** conceptualization (equal), data curation (lead), formal analysis (lead), funding acquisition (equal), methodology (equal), visualization (lead), writing – original draft (lead), writing – review and editing (equal). **Ariana E. Tao:** data curation (equal), project administration (lead), supervision (lead), writing – original draft (lead), writing – review and editing (equal). **Brieyona C. Reaves:** conceptualization (equal), funding acquisition (equal), methodology (equal), project administration (supporting), supervision (equal), writing – review and editing (equal). **Krystal A. Rivera:** data curation (supporting), project administration (supporting), writing – review and editing (supporting). **Lauren K. Jones:** data curation (supporting), project administration (supporting), writing – review and editing (supporting). **Rita R. Ravichandar:** data curation (supporting), project administration (supporting), writing – review and editing (supporting). **Dennis Yi‐Shin Kuo:** resources (equal), writing – review and editing (equal). **Rafi Kabarriti:** resources (equal), writing – review and editing (equal). **Alexander I. Sankin:** resources (equal), writing – review and editing (equal). **Ahmed A. Aboumohamed:** resources (equal), writing – review and editing (equal). **Kara L. Watts:** resources (equal), writing – review and editing (equal). **Damara N. Gutnick:** conceptualization (supporting), writing – original draft (supporting), writing – review and editing (equal). **Ellen Miller‐Sonet:** conceptualization (equal), funding acquisition (equal), methodology (equal), writing – original draft (lead), writing – review and editing (equal).

## Conflicts of Interest

The authors declare no conflicts of interest.

## Data Availability

Data will be made available upon written request to the corresponding author and will be shared in compliance with institutional data‐sharing policies.
